# Mutation Analysis of Consanguineous Moroccan Patients with Parkinson’s Disease Combining Microarray and Gene Panel

**DOI:** 10.3389/fneur.2017.00567

**Published:** 2017-10-31

**Authors:** Ahmed Bouhouche, Christelle Tesson, Wafaa Regragui, Mounia Rahmani, Valérie Drouet, Houyam Tibar, Zouhayr Souirti, Rafiqua Ben El Haj, Naima Bouslam, Mohamed Yahyaoui, Alexis Brice, Ali Benomar, Suzanne Lesage

**Affiliations:** ^1^Research Team in Neurology and Neurogenetics, Faculty of Medicine and Pharmacy, Genomics Center of Human Pathologies, University Mohammed V, Rabat, Morocco; ^2^Sorbonne Universités, UPMC Université Paris 6 UMR_S 1127, INSERM U 1127, CNRS UMR 7225, Institut du Cerveau et de la Moelle épinière, ICM, Paris, France; ^3^Clinical Neurosciences Laboratory, Faculty of Medicine and Pharmacy, Sidi Mohamed Ben Abdellah University, Fez, Morocco

**Keywords:** Parkinson’s disease, Moroccan patients, consanguinity, chromosomal microarray analysis, next-generation sequencing gene panel

## Abstract

During the last two decades, 15 different genes have been reported to be responsible for the monogenic form of Parkinson’s disease (PD), representing a worldwide frequency of 5–10%. Among them, 10 genes have been associated with autosomal recessive PD, with *PRKN* and *PINK1* being the most frequent. In a cohort of 145 unrelated Moroccan PD patients enrolled since 2013, 19 patients were born from a consanguineous marriage, of which 15 were isolated cases and 4 familial. One patient was homozygous for the common *LRRK2* G2019S mutation and the 18 others who did not carry this mutation were screened for exon rearrangements in the *PRKN* gene using Affymetrix Cytoscan HD microarray. Two patients were determined homozygous for *PRKN* exon-deletions, while another patient presented with compound heterozygous inheritance (3/18, 17%). Two other patients showed a region of homozygosity covering the 1p36.12 locus and were sequenced for the candidate *PINK1* gene, which revealed two homozygous point mutations: the known Q456X mutation in exon 7 and a novel L539F variation in exon 8. The 13 remaining patients were subjected to next-generation sequencing (NGS) that targeted a panel of 22 PD-causing genes and overlapping phenotypes. NGS data showed that two unrelated consanguineous patients with juvenile-onset PD (12 and 13 years) carried the same homozygous stop mutation W258X in the *ATP13A2* gene, possibly resulting from a founder effect; and one patient with late onset (76 years) carried a novel heterozygous frameshift mutation in *SYNJ1*. Clinical analysis showed that patients with the *ATP13A2* mutation developed juvenile-onset PD with a severe phenotype, whereas patients having either *PRKN* or *PINK1* mutations displayed early-onset PD with a relatively mild phenotype. By identifying pathogenic mutations in 45% (8/18) of our consanguineous Moroccan PD series, we demonstrate that the combination of chromosomal microarray analysis and NGS is a powerful approach to pinpoint the genetic bases of autosomal recessive PD, particularly in countries with a high rate of consanguinity.

## Introduction

Parkinson’s disease (PD; MIM #168601) is a common neurodegenerative disorder with a prevalence of >1% in populations over 60 years of age (1). PD is clinically characterized by rigidity, bradykinesia, tremor, and postural instability, and may be accompanied with dementia and depression (2, 3). The disease etiology is likely to be multifactorial, involving complex interactions between genetic and environmental factors. In the past 20 years, genetic studies of PD families have provided strong support for the hypothesis that PD has a significant genetic component. To date, 13 genes have been described for hereditary PD (4, 5), and at least 10 of these genes are associated with autosomal recessive (AR) forms of PD. Although mutations in PARK2 (*PRKN*; MIM #602544), PARK6 (*PINK1*; MIM #605909), and PARK7 (*DJ1*, MIM #606324) are infrequent in the PD population, they are responsible for a majority of early-onset PD and are usually known to cause typical PD with indistinguishable clinical signs (1, 4, 6). Moreover, the other, more rare mutations, including PARK9 (*ATP13A2*; MIM #610513), PARK14 (*PLA2G6*; MIM #612953), PARK15 (*FBXO7*; MIM #260300), PARK19 (*DNAJC6*; MIM #615528), PARK20 (*SYNJ1*; MIM #615530), PARK23 (*VPS13C*; MIM #616840), and *PODXL* (MIM #602632) have been implicated in parkinsonism characterized by juvenile onset and atypical clinical signs (5, 7–10).

*PRKN*, located on chromosome 6q26, is one of the largest human genes spanning 1.38 Mb with 12 exons and large intronic regions (11). Mutations in this gene explain up to 50% of AR PD and about 15% of sporadic cases with early onset (12, 13). A large number of mutations have been identified in all populations studied, regardless of ethnic origin, including exon rearrangements and point mutations. *PINK1* contains eight exons that span 1.8 kb and encode a 581-amino-acid serine/threonine kinase protein. This protein exhibits an N-terminal mitochondrial targeting sequence, a putative transmembrane anchor as well as a C-terminal kinase domain (14, 15). It was noticed that the mutational hotspot is found in exon 7. More than 70 mutations have been reported in *PINK1*; two-thirds of which are loss-of-function mutations affecting its kinase domain (16). *DJ1* is composed of eight exons spanning 24 kb and encodes a 189-amino-acid protein (17, 18). The causative PD mutations identified in *DJ1* are mainly point and structural mutations resulting in loss of protein function (17, 19). All *PRKN, PINK1*, and *DJ1* mutation carriers present with similar clinical features that are practically indistinguishable from idiopathic forms of PD, including a good response to levodopa with a tendency to develop levodopa-induced dyskinesia and a slow progression.

In addition, mutations in *ATP13A2* cause a juvenile parkinsonism characterized by a rapid progression, supranuclear gaze palsy, pyramidal signs, and dementia (4, 20). Mutations in *PLA2G6* display both early-onset and juvenile forms of recessively inherited atypical parkinsonism. The early-onset form is associated with levodopa-responsive dystonia-parkinsonism, pyramidal signs, cognitive dysfunction, and Lewy body disease accumulation in the brain, whereas the juvenile one is distinguished by dystonia, cerebellar ataxia, spasticity in all limbs, and cognitive decline (21). Mutations in *FBXO7* are also responsible for a juvenile form that presents with early dystonia and pyramidal signs (22). Furthermore, the gene *DNAJC6* encodes for the HSP40 Auxilin protein. Mutations in *DNAJC6* induce an early-onset parkinsonian-pyramidal phenotype with rapid progression and a poor response to levodopa. Patients with these mutations can also show juvenile parkinsonism with a relatively slow disease progression (7, 23, 24). Also responsible for early-onset PD, *SYNJ1* mutations cause atypical parkinsonism with a number of features, such as dystonia, oculomotor apraxia, dementia, and seizures (25–27). *VPS13C* is a multi-exonic gene (86 exons) responsible for early-onset parkinsonism, and it is associated with a severe phenotype characterized by rapid progression, cognitive deterioration, and a widely distributed presence of Lewy bodies (9, 23). Recently, a homozygous frameshift mutation in the *PODXL* gene was described as a causal factor for juvenile parkinsonism. Although these data are currently insufficient to support this assertion, the clinical features appear similar to those of classic PD (10).

In the present study, we analyzed the genetic bases of a series of consanguineous PD patients from Morocco by combining chromosomal microarray analysis (CMA) and gene panel next-generation sequencing (NGS) to target 22 genes associated with PD and overlapping phenotypes.

## Patients and Methods

### Patients

From 2013 to 2016, a total of 145 Moroccan PD patients were enrolled at the Movement Disorder Unit of the Department of Neurology (Specialties Hospital, Rabat, Morocco). Clinical Diagnosis of PD was made using the United Kingdom Parkinson’s Disease Society Brain Bank criteria (28). Patients were submitted to a structured clinical interview as described previously (29). Nineteen of 145 patients (13%) were born from a consanguineous marriage, of which 15 were isolated cases and 4 were familial cases. Genomic DNA was extracted from peripheral blood leukocytes using Isolate II Genomic DNA kit from Bioline. This study was approved by the Biomedical Research Ethics Committee of the Medical School of Rabat (CERB) and written informed consent was obtained from all subjects in accordance with the Declaration of Helsinki.

### Genetic Analysis

To assess the 19 consanguineous PD patients selected for this study, we first sequenced exon 41 of Leucine-rich repeat kinase 2 gene (*LRRK2*; MIM #609007) to screen for the G2019S mutation that was reported to represent 41% of Moroccan PD patients (29).

### Chromosomal Microarray Analysis

DNA samples of patients negative for the G2019S mutation were then screened for exon rearrangements using Affymetrix Cytoscan HD microarray according to the manufacturer’s protocol. With a median inter-marker distance of 500–600 bp, CytoScan HD offers the highest physical coverage of the genome for detecting human chromosomal abnormalities. Indeed, these chips include 750,000 single-nucleotide polymorphism (SNP) and 2.6 million copy number variation (CNV) markers that enable high-resolution (25–50 kb resolution) detection of CNVs, region of homozygosity (ROH), uniparental disomy, and low-level mosaicism. Briefly, 250 ng of DNA samples were digested with *Nsp1*, amplified with TITANIUM Taq DNA polymerase (Clontech, Mountain View, CA, USA), fragmented with Affymetrix fragmentation reagent, and labeled with biotin end-labeled nucleotides. DNAs were hybridized to the microarrays for 16 h, washed and stained on the GeneChip Fluidics Station 450, and scanned on the GeneChip Scanner 3000 7G (Affymetrix). Data analysis was performed using Chromosome Analysis Suite software version 1.2.2 (Affymetrix). Data were considered significant only when they met the quality control criteria set by the manufacturer [the Median Absolute Pairwise Difference scores (MAPD) < 0.25, the Waviness Standard Deviation (WSD) < 0.12, and the SNP Quality Control (SNP-QC) > 0.15].

### DNA Sanger Sequencing

DNAs of patients with ROH covering the 1p36.12 locus were sequenced for *PINK1*. All eight of the coding exons and intron–exon boundaries of *PRKN* and *PINK1* were polymerase chain reaction (PCR) amplified and the PCR products were sequenced using Big Dye Terminator Cycle Ready Reaction 3.1 Kits and an ABI 3130xl automated sequencer for the patients and the 96 controls. The collected sequence data were analyzed using SeqScape2.1 software (Applied Biosystems, Foster City, CA, USA).

### Next-Generation Sequencing (NGS) Target Gene Panel and Validation

We designed an NGS-based screening of the 22 currently most prevalent parkinsonism-associated genes (Table S1 in Supplementary Material). The custom Design KAPPA Library Preparation Kit (Roche) was used to capture all exons, intron–exon boundaries, 5′- and 3′-UTR sequences and 10-bp flanking sequences of target genes (RefSeq database, hg19 assembly). Specific probes for NGS target enrichment were designed using NimbleDesign^1^ software and amplicon length varied between 250 and 500 bp. Runs were performed on Illumina MiSeq sequencer. The assay was performed according to the manufacturer’s recommended protocol. Variants were prioritized based on the following criteria: frequencies <0.01% in public databases (ExAC/GnomAD) and our in-house database of 500 exomes, nucleotide and amino-acid conservation (based on alignments), relation of the gene to disease (per family), and inheritance pattern. All reported variants were confirmed by Sanger sequencing.

### Bioinformatics Analysis of Gene Panel Data

Human reference genome UCSC hg19 was used for sequence alignment and variant calling with the Burrows-Wheeler Aligner (BWA)^2^ (30) and the Genome Analysis Toolkit (GATK)^3^ (31). PCR duplicates were removed prior to variant calling using Picard^4^ software. Variants were annotated with ANNOVAR software (32). The mean coverage was 993× (range 594–1241×), and the mean percent coverage at 30× was 98.7% (range 96.5–99.6%) for all individuals tested. Targeted exons with a coverage less than 30 reads were screened subsequently by Sanger sequencing. Analyses were performed using custom Polyweb software (Paris Descartes, France).

## Results

Of the 145 PD patients recruited during the 4-year period, 19 individuals were born from a consanguineous marriage, which represents an inbreeding rate of 13%. Of them, seven patients were males (37%) and four had a positive family history of PD (21%). The mean age at examination was 56.2 years (range 19–84) and the mean age at onset was 47.4 (range 12–77) years. Sanger screening for the *LRRK2* exon 41 showed the G2019S mutation in the homozygous state for one patient without family history of the disease (Table 1, patient ID 3332). The clinical phenotype of this patient, whose disease onset was recorded at 48 years and who had a disease duration of 9 years, is no different from the heterozygous G2019S carriers described previously (29). Because the G2019S mutation is very common in Morocco, this patient will be included in a large series of G2019S carriers to be analyzed for the dopamine metabolism genes in order to determine their effects on age of onset, clinical phenotype and response to treatment.

**Table 1:** Clinical features of the nine consanguineous Moroccan PD patients with gene mutation.

Patients ID	3332	3022	3868	3467	3020	3158	3468	3223	3528
Mutation, gene	G2019S, Hmz LRRK2	W258X, Hmz ATP13A2	W258X, Hmz ATP13A2	S552Ffs*5, Htz SYNJ1	Ex 3-4del/Ex3-7del, Htz PARK2	Ex9del, Hmz PARK2	Ex6-7del, Hmz PARK2	L539F, Hmz PINK1	Q456X, Hmz PINK1
Isolated/familial case	IC	IC	IC	FC	IC	IC	FC	IC	FC
Age at onset	48	12	13	76	17	43	39	54	42
Disease duration	9	7	12	2	35	6	15	4	20
Initial symptom	Tremor	Akinesia, Dystonia, Swalowing	Bradykinesia	Tremor, Bradykinesia	Tremor	Bradykinesia	Tremor	Tremor, Bradykinesia	Tremor
Clinical Form	Trembling	Akinetic-Rigid	Akinetic-Rigid	Mixed	Trembling	Mixed	Mixed	Mixed	Mixed
Resting tremor	−	−	−	+	+	+	+	+	+
Akinesia	−	+	+	+	+	+	+	−	+
Rigidity	+	+	+	+	+	+	−	+	−
Dystonia	−	+	−	−	+	−	−	−	−
Gait impairment	−	+	+	+	+	+	+	+	−
Postural instability	−	+	+	+	−	+	−	−	−
UPDRS III	3	16	24	8	15	14	20	13	9
H-Y score	3	4	4	3	3	1	2	3	2.5
Motor fluctuation	−	+	−	−	−	−	−	+	+
Levodopa-induced dyskinesia	−	+	−	−	−	−	−	−	+
Levodopa equivalent dose	400	400	Sifrol1c/j	400	Artane 5 mg 4cp/j	300	150	1000	400
Urinary dysfunction	+	−	−	−	−	+	+	−	+
Orthostatic HypoTA	+	−	−	−	−	+	−	−	+
Pain	−	−	−	−	−	−	−	−	−
Constipation	−	−	−	+	−	+	−	−	−
Sleep disorder	−	−	−	+	−	−	−	+	−
Psychiatric features	−	−	−	−	−	−	−	+	−
Cognitive decline	+	−	+	−	−	+	−	−	+

*Hmz, homozygous; Htz, heterozygous; W, woman; M, male*.

**Figure 1:**
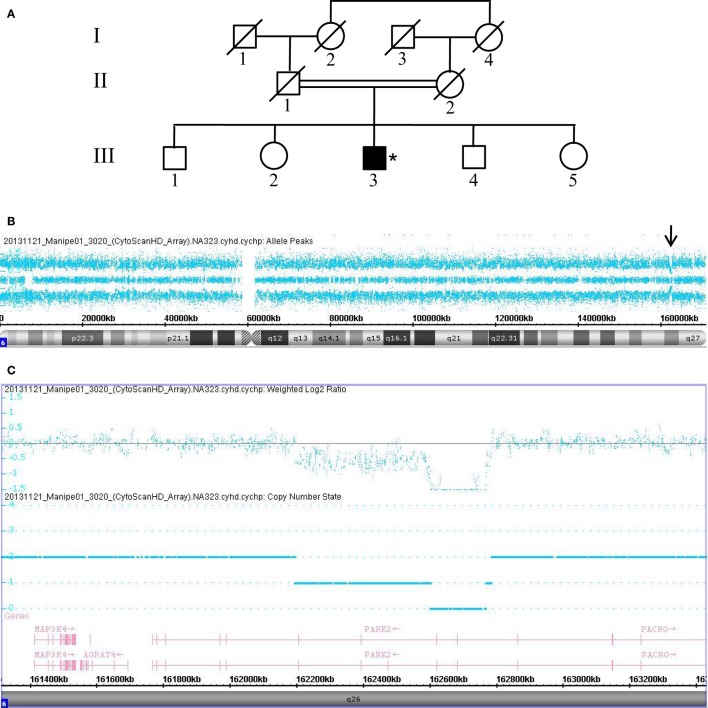
Chromosomal microarray analysis showing two compound heterozygous deletions in *PRKN*. **(A)** Pedigree of patient family, (*) indicates proband. **(B)** Allele-difference plot, arrow indicates CNV at position 6q26. **(C)** Weighted log_2_ ratio and copy number state plots. CNV, copy number variation.

The remaining 18 patients negative for the *LRRK2* G2019S mutation were subjected to high-resolution CMA using an Affymterix platform and CytoscanHD microarrays, which revealed microdeletion chromosomal region 6q26 of the *PRKN* in 3 of the 18 consanguineous PD patients (16.7%). The deletions were determined to be homozygous in two patients and compound heterozygous in one patient. Data collected from Patient 3020, who carries a compound heterozygous deletion, are shown in Figure 1. This male patient (III.3) is a sibling to four other, unaffected children born from a common consanguineous marriage of the first degree (Figure 1A). Despite the patient’s consanguineous heritage, the allele-difference plot did not show ROH at chromosome 6q (Figure 1B). The weighted log_2_ ratio and copy-number-state plots of chromosomal region 6q26 show two heterozygous *PRKN* deletions of 166 and 587 kb encompassing exons 3–4 and exons 3–7, respectively (Figure 1C). The centromeric and telomeric break points of the smallest deletion were located at positions 162.600.881 and 162.767.227, and the break points of the largest deletion were located at positions 162.195.309 and 162.783.147, respectively. Absence of ROH at region 6q26 suggests that the two deletions were inherited from two different ancestors, and thus the patient’s father (II.1) and mother (II.2) must carry one of the two mutations in the heterozygous state.

**Figure 2:**
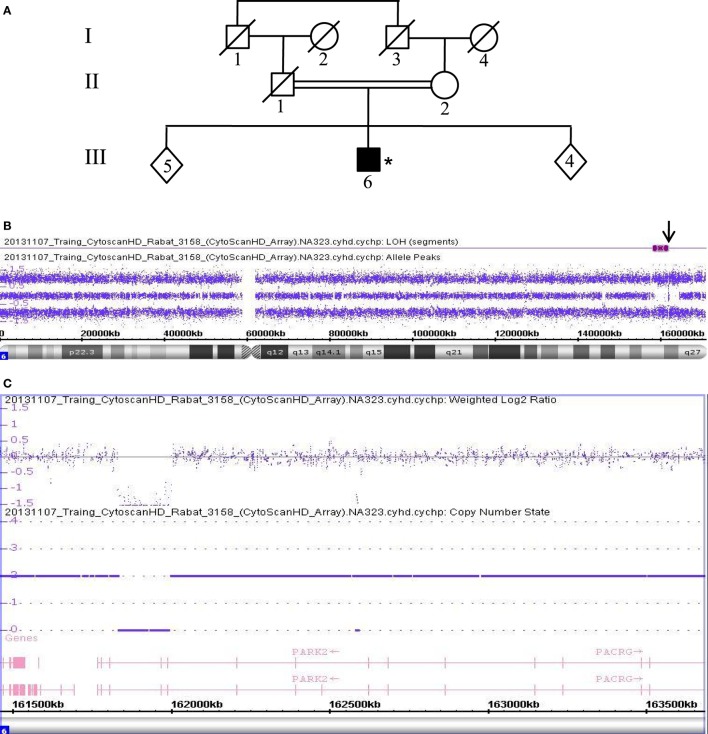
Chromosomal microarray analysis showing a homozygous deletion in *PRKN* in Patient 3158. **(A)** Pedigree of patient family, (*) indicates proband. **(B)** Allele-difference plot, arrow indicates ROH and CNV at position 6q26. **(C)** Weighted log_2_ ratio and copy number state plots. CNV, copy number variation; ROH, region of homozygosity.

In addition, patient 3158 (III.6), a 49-year-old male, was also born from a consanguineous marriage of the first degree (Figure 2A). The allele-difference plot shows a 6.5 Mb ROH at chromosome 6q (Figure 2B). The weighted log_2_ ratio and copy-number-state plots of the 6q26 region show a homozygous deletion of 154 kb that encompasses exon 9 of the *PRKN* gene (Figure 2C). The centromeric break point of the deletion was located within intron 9 (161.835.769), and the telomeric break point was located in intron 7 (161.990.516).

**Figure 3:**
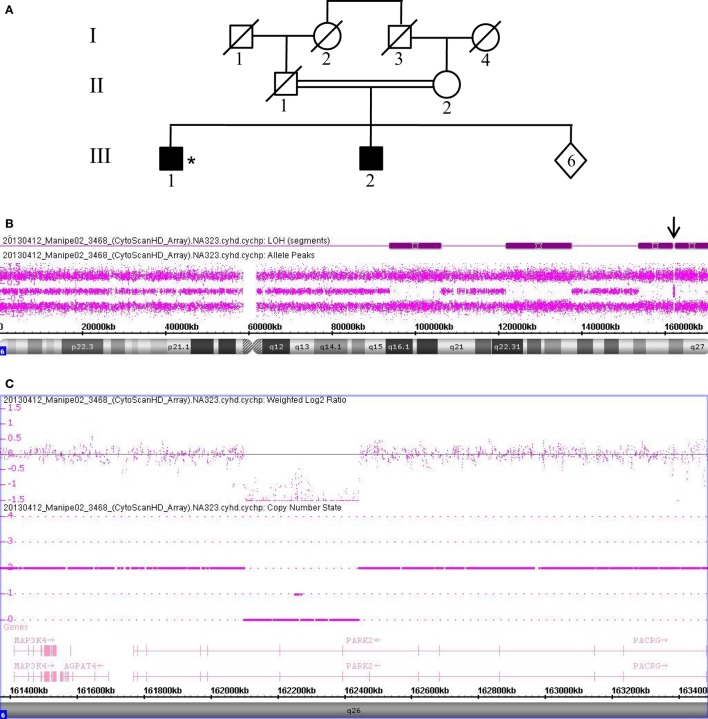
Chromosomal microarray analysis showing a homozygous deletion in *PRKN* in Patient 3468. **(A)** Pedigree of patient family, (*) indicates proband. **(B)** Allele-difference plot. Arrow indicates CNV at position 6q26. **(C)** Weighted log_2_ ratio and copy number state plots. CNV, copy number variation.

Data collected from patient 3468, who is also homozygous for a *PRKN* gene deletion, are shown in Figure 3. This patient (III.1) was born from a first-degree consanguineous marriage and has a brother known to have PD (Figure 3A). The allele-difference plot shows three long, contiguous stretches of homozygosity at chromosome 6q: (1) 12.49 Mb from 6q16.1-q21, (2) 15.79 Mb from 6q22.31-q23.3, and (3) 17.2 Mb from 6q25.2-qter (Figure 3B). The weighted log_2_ ratio and copy-number-state plots of the 6q26 region show a homozygous deletion of 339kb encompassing exons 6 and 7 of the *PRKN* gene (Figure 3C). While the centromeric break point of the deletion was located within intron 7 (162.101.291), the telomeric break point was located in intron 5 (162.441.087).

While Patients 3158 and 3468 presented with early-onset PD with mixed phenotypes, patient 3020 presented with juvenile-onset disease with a phenotype of tremors and dystonia. The three patients were improved by very low doses of antiparkinsonian drugs without motor fluctuations or dyskinesia, even after up to 35 years of disease duration in Patient 3020 (Table 1).

**Figure 4:**
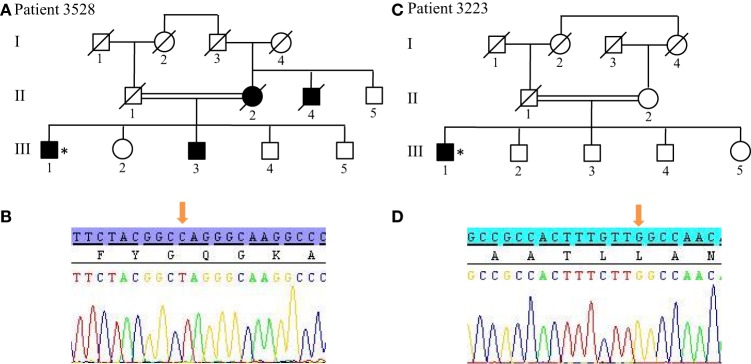
*PINK1* mutations in patients with ROH at 1q position. **(A,B)** Pedigree of Patient 3528 family and Sanger sequencing plot of *PINK1* showing the Q456X mutation in homozygous state. **(C,D)** Pedigree of Patient 3223 family and Sanger sequencing plot of *PINK1* showing the p.L539F mutation in homozygous state. (*) indicates proband and arrows indicate the mutation position. ROH, region of homozygosity.

For patients without *PRKN* exon deletions, the high-resolution karyotype was normal and no CNV was identified within the known PD loci. However, because Patients 3528 and 3223 showed ROH covering the 1p36.12 locus, they were also sequenced for the candidate *PINK1* gene. Results revealed two homozygous point mutations: Q456X (c.1366C > T) (Figures 4A,B) mutation in exon 7 and novel L539F (c.1617G > C) variation in exon 8 (Figures 4C,D). The patient with the Q456X mutation showed an early-onset disease, mixed phenotype without balance impairment after 20 years of disease duration, dyskinesia with very low dose levodopa therapy (400 mg/day), and a mild degree of cognitive impairment. Interestingly, the patient with the L539F mutation manifested parkinsonism at 50 years of age with high doses of levodopa (1000mg/day) after 4 years of disease progression (Table 1).

### NGS Panel and Validation Results

**Figure 5:**
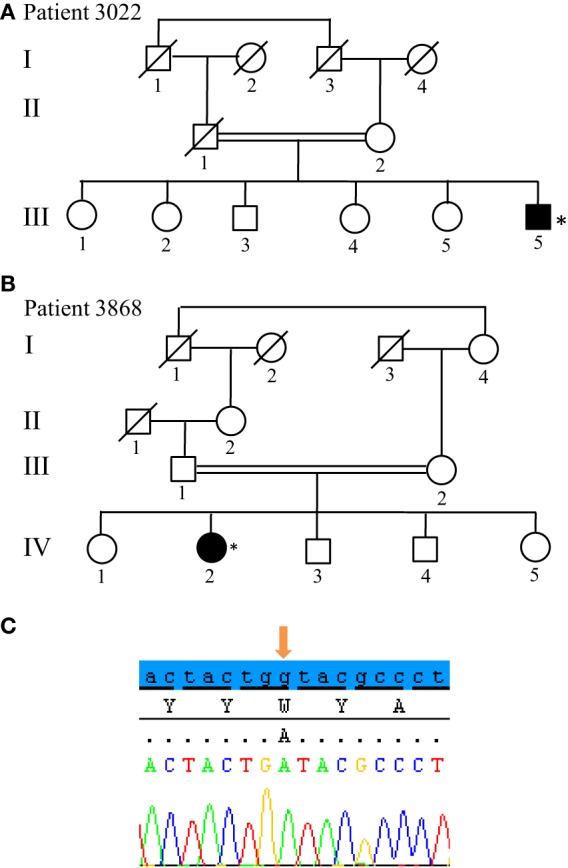
Novel non-sense mutation W258X in *ATP13A2* found in two patients. **(A,B)** Pedigree of Patients 3022 and 3868, respectively. **(C)** Sanger sequencing plot of *ATP13A2* showing the W258X mutation at homozygous state. (*) indicates proband and arrows indicate the mutation position.

**Figure 6:**
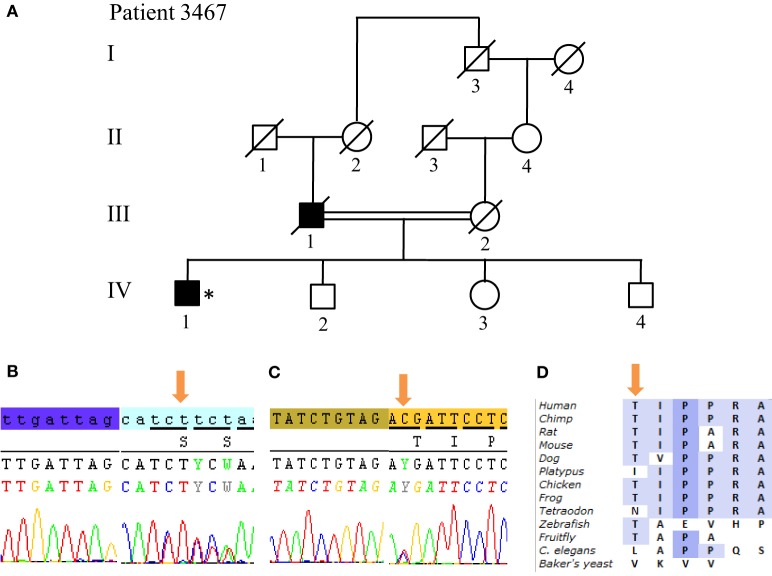
*SYNJ1* mutations found in Patient 3467 at compound heterozygous state. **(A)** Pedigree of patient family. **(B)** Sanger sequencing plot of the mutation S552Ffs*5. **(C)** Sanger sequencing plot of the mutation T1236M. **(D)** Conservation of amino-acid Thr among various species. (*) indicates proband and arrows indicate the mutation position.

From the 13 patients analyzed by NGS, we identified causative mutations affecting three individuals. In patients 3022 and 3868, we identified the same p.W258X (c.774 G > A) stop mutation in *ATP13A2* (Figure 5). For individual 3022, a homozygous mutation in *ATP13A2* was suspected, given that a loss of heterozygosity was detected at this locus on microarray analysis. Patients with the W258X mutation in *ATP13A2* showed a juvenile-onset disease (12 and 13 years), an akinetic-rigid phenotype with shuffling gait and postural instability. Patient 3022 had dystonia and swallowing difficulties in the very onset of the disease. He also displayed motor fluctuations and dyskinesia at very low doses of dopaminergic drugs (400 mg of levodopa equivalent daily dose) after 7 years of disease duration. Patient 3868 presented with some cognitive decline 12 years after disease onset. No other non-motor symptoms are present in both patients (Table 1). Finally, we identified in Patient 3467 two heterozygous mutations in *SYNJ1*: a p.S552Ffs*5 (c.1655delG) frameshift mutation and a p.T1236M (c.3707 C > T) missense variant (Figure 6). The missense variant is most likely benign as it is reported at relatively high frequency in the ExAC database (rs145937537, minor allele frequency in all populations: 0.0015 and in African population: 0.0017) but was not reported in the homozygous state. It is predicted benign by 6 of 7 *in silico* pathogenicity prediction tools (SIFT,^5^ PolyPhen-2,^6^ Mutation Taster,^7^ LRT,^8^ and FATHMM Radial SVM, LR score).^9^ This patient was a 76-year-old male born from consanguineous parents (Figure 6) with a slowly progressive form of typical PD, and a phenotype of akinetic-rigidity, tremors, postural instability, sleep disorders, and constipation. He was treated by very low doses of levodopa and displayed no apparent cognitive impairment. His father was diagnosed with parkinsonism at the age of 63 years, and he died 10 years later while still walking independently.

The remaining 10 patients did not have any identifiable mutations in the targeted PD-associated genes. These patients displayed a mean age of onset of 55.6 years [42–77]. Their clinical phenotype ranged between akinetic-rigid to mixed form without dystonia but with postural instability in up to 70% of cases after 5.7 years [1–19] of disease duration, on average. Motor fluctuations are present in 5 of 10 patients, even those with short disease duration of 3 or 4 years, whereas the dyskinesia phenotype is only observed in two patients that each have had PD for more than 10 years. All patients displayed at least two non-motor signs; urinary dysfunction being the most frequent (Table S2 in Supplementary Material).

## Discussion

Of the *PRKN* mutations reported so far, large deletions are the most frequent and can be present in either homozygous or compound heterozygous states (16, 33, 34). However, due to limitations of the multiplex ligation-dependent probe amplification (MLPA) and quantitative PCR methods used commonly, only few studies have determined the size and break point locations of the rearrangements. We overcame this issue by employing high-resolution CMA, which allows for rapid and effective detection of CNV and their break point locations. Through use of Cytoscan HD arrays, the screening of 18 consanguineous, Moroccan PD patients revealed four deletions in three probands. Our results show a rearrangement frequency of 16.7% in consanguineous Moroccan PD patients. The deletions were determined to be homozygous in two patients and compound heterozygous in one patient. All the rearrangements observed in our sample were deletions located between exons 2 and 9, and their break point locations appear to be unique. In patient 3020, although the two deletions were inherited from two individuals of the same, highly consanguineous family, the break point locations of their intron were different. These findings suggest that the deletions were independent and recurrent events, which confirms previous studies reporting that most *PRKN* gene deletions occurred between exons 2 and 8. This region of *PRKN* comprises the FRA6E center and is considered a deletion hotspot because it contains certain microhomology sequences that have been frequently implicated in the main rearrangement process (33, 35, 36).

Moreover, in addition to the detection of CNV, the CMA method enables detection of ROH, which is effective for identifying candidate genes of recessive diseases. Indeed, two patients without *PRKN* CNV showed ROH at the 1p36 locus and had mutations in the *PINK1* gene. These mutations consisted of the already known Q456X mutation in exon 7 and a new L539F variant in exon 8; the latter of which is located in the C-terminal domain of the *PINK1* protein that we have already shown to be probably pathogenic (37).

Furthermore, we demonstrate the feasibility of NGS as a research and potential diagnostic tool for patients with parkinsonism. For instance, we identified the same homozygous nonsense mutation of p.W258X in *ATP13A2* in two unrelated consanguineous patients, probably resulting from a common founder. This mutation falls into an ROH at chromosome 1p, detected in one patient. We also found two different heterozygous variants in *SYNJ1* in one patient: a novel frameshift mutation and a missense change that is likely benign.

Clinically, our patients with mutations in *PRKN* and *PINK1* presented with a classical PD phenotype with early onset, good response to levodopa, and benign course as previously reported in the literature (12). It should be noted that the patient sharing two compound heterozygous deletions in *PRKN* with a juvenile onset, tremoric phenotype, and dystonia, did not exhibit non-motor symptoms after 35 years of disease duration. Also, for the patient with the *PINK1* stop mutation, postural instability was not seen after 20 years of disease duration. Otherwise, mutations in *ATP13A2* was reported to cause Kufor–Rakeb syndrome with juvenile onset and atypical clinical features including pyramidal signs, dystonia, cognitive decline, supranuclear gaze palsy (38) and may become unresponsive to levodopa as the disease progresses (39). Our patients who had the same novel R258X mutation had few atypical signs, namely dystonia and cognitive impairment, but one patient also displayed bulbar symptoms at the onset of the disease. These bulbar symptoms can be explained by either bilateral pyramidal signs (pseudo-bulbar syndrome). Furthermore, mutations in *SYNJ1* have been reported previously to cause AR PD with symptom onset occurring after about 30 years of age and with disease progression ranging from severe to stable. Also, it has been observed that *SYNJ1* mutations induce severe dyskinesia with generalized seizures in childhood, dystonia, developmental delay, cognitive impairment, and oculomotor disturbances even when coupled with low-dose levodopa therapy (25, 40). However, we report here a male patient heterozygous for a novel frameshift mutation in *SYNJ1* who displays a late-onset, slowly progressive form of typical PD and responds well to low-dose levodopa therapy without apparent cognitive impairment. Though unavailable for examination, his father also had parkinsonism, who by the patient’s accounts followed a similarly benign course. This case is thus consistent with a pseudo-dominant pattern of inheritance or recessive inheritance in compound association with another heterozygous mutation in a gene that not exists in the panel used.

In conclusion, this study provides evidence that supports the power and efficiency of the high-resolution CMA method for uncovering micro-rearrangements in the *PRKN* gene for patients with AR PD. By focusing on patient populations with a high prevalence of consanguinity in Morocco, we also demonstrate the potential of this approach to be used a first-line diagnostic test. Furthermore, the combination of CMA with NGS provides an important understanding of the genetic bases of AR PD.

## Ethics Statement

This study was approved by the biomedical research ethics committee of the Medical School of Rabat (CERB) and written informed consent was obtained from all subjects in accordance with the Declaration of Helsinki.

## Author Contributions

WR, HT, MR, and ZS phenotyped patients. RB and NB performed DNA extraction and banking. RB performed Sanger Sequencing. ABo performed microarray analysis in the Medical School of Rabat. CT and VD performed NGS analyses at the ICM platform. SL and AB supervised the work at the ICM institute. ABo, ABe, and MY supervised the work and obtained funding support. ABo and RB wrote the manuscript. All authors edited the final version of the manuscript.

## Conflict of Interest Statement

The authors declare that the research was conducted in the absence of any commercial or financial relationships that could be construed as a potential conflict of interest.

## Footnotes

^1^https://design.nimblegen.com/nimbledesign/app/.

^2^http://bio-bwa.sourceforge.net/.

^3^http://www.acronymfinder.com/Genome-Analysis-Toolkit-(software)-(GATK).html.

^4^http://picard.sourceforge.net/.

^5^http://sift.jcvi.org/www/SIFT_enst_submit.html.

^6^http://genetics.bwh.harvard.edu/pph2/index.shtml.

^7^http://www.mutationtaster.org/.

^8^http://www.genetics.wustl.edu/jflab/lrt_query.html.

^9^http://fathmm.biocompute.org.uk/.
