# Supplementary material for: Mutation Analysis of Consanguineous Moroccan Patients with Parkinson’s Disease Combining Microarray and Gene Panel
Source: Front Neurol. 2017 Oct 31;8:567. doi: 10.3389/fneur.2017.00567 (PMC5674924; doi:10.3389/fneur.2017.00567)
Supplement: Supplementary file 2 [file Table_2.DOC]

**Supplementary Table**: Clinical Features of the 10 consanguineous Moroccan PD patients without gene mutation

| **Patients** | **3446** | **3472** | **3536** | **3627** | **3668** | **3680** | **3691** | **3786** | **3844** | **3852** |
| --- | --- | --- | --- | --- | --- | --- | --- | --- | --- | --- |
| Age at onset | 47 | 44 | 42 | 51 | 63 | 51 | 56 | 62 | 63 | 77 |
| Disease duration | 10 | 4 | 4 | 19 | 2 | 5 | 2 | 3 | 1 | 7 |
| Clinical Form | Akinetic-Rigid | Akinetic-Rigid | Akinetic-Rigid | Akinetic-Rigid | Mixed | Akinetic-Rigid | Mixed | Mixed | Mixed | Mixed |
| Resting tremor | - | - | - | - | + | - | + | + | + | + |
| Akinesia | + | + | + | + | + | + | + | + | + | - |
| Rigidity | + | + | + | + | + | + | + | + | + | + |
| Dystonia | - | + | - | - | - | - | - | - | - | - |
| Gait Impairment | + | - | - | - | + | - | + | - | + | - |
| Postural instability | + | + | + | + | + | + | - | - | + | - |
| UPDRS III | 26.5 | 6 | 8 | 29 | 13 | 15 | 6 | 18 | 42 | 26 |
| H-Y Score | 3 | 3 | 2 | 1 | 1.5 | 1.5 | 1 | 2 | 1.5 | 2 |
| Motor Fluctuation | + | + | + | + | - | - | - | + | - | - |
| Levodopa induced dyskinesia | + | - | - | + | - | - | - | - | - | - |
| Levodopa equivalent dose | 2200 | 850 | 1200 | 600 | 200 | 450 | 100 | 500 | 150 | 0 |
| Urinary dysfunction | NA | + | + | + | + | - | + | + | + | + |
| Orthostatic HypoTA | NA | - | + | - | + | - | - | - | + | - |
| Pain | + | - | + | + | - | - | - | - | - | + |
| Constipation | + | - | + | + | + | + | - | - | - | - |
| Sleep disorder | + | + | + | - | - | - | + | + | + | - |
| Psychiatric features | + | - | + | - | - | + | + | + | - | - |
| Cognitive decline | + | + | - | - | - | - | - | - | + | - |

B : Bradykinesia; T : Tremor; NA : not attributed
